# *PDCD1* genes may protect against extraocular manifestations in Chinese Han patients with Vogt-Koyanagi-Harada syndrome

**Published:** 2009-02-20

**Authors:** Qianli Meng, Xiaoli Liu, Peizeng Yang, Shengping Hou, Liping Du, Hongyan Zhou, Aize Kijlstra

**Affiliations:** 1 Zhongshan Ophthalmic Center, Sun Yat-sen University, Uveitis Study Center of Sun Yat-sen University and International Uveitis Study Laboratory of Guangdong Province, State Key Laboratory of Ophthalmology of Sun Yat-sen University, Guangzhou, P.R. China; 2 The First Affiliated Hospital, Chongqing Medical University, Chongqing key laboratory of ophthalmology, Chongqing, P. R. China; 3 Eye Research Institute Maastricht, Department of Ophthalmology, University Hospital Maastricht, Maastricht, the Netherlands; 4 Animal Sciences Group, Wageningen UR, Lelystad, the Netherlands

## Abstract

**Purpose:**

To analyze the potential association of programmed cell death 1 (*PDCD1*) with Vogt-Koyanagi-Harada (VKH) syndrome in a Chinese Han population.

**Methods:**

Three single nucleotide polymorphism (SNPs), PD-1.3G/A, PD-1.5C/T, and PD-1.6G/A, were genotyped in 247 VKH patients and 289 age-, sex-, and ethnically-matched healthy controls using polymerase chain reaction-restriction fragment length polymorphism (PCR-RFLP) assay. The associations of genotypes and alleles with VKH syndrome were analyzed.

**Results:**

All genotype distributions in healthy controls were in Hardy–Weinberg equilibrium. The genotype and allele frequencies of PD-1.3, PD-1.5, and PD-1.6 were not different between patients with VKH syndrome and healthy controls. No significant difference was observed according to the status of human leukocyte antigen (HLA)-DR4 and HLA-DRw53. Compared to the controls, lower frequencies of the PD-1.5C genotype and allele frequencies were observed in VKH patients with extraocular findings.

**Conclusions:**

PD-1.3 and PD-1.6 polymorphisms are not associated with the susceptibility to VKH syndrome in the Chinese Han population. However, PD-1.5 may be negatively associated with the occurrence of extraocular manifestations of VKH syndrome.

## Introduction

Vogt-Koyanagi-Harada (VKH) syndrome is an autoimmune disease characterized by a bilateral granulomatous panuveitis and systemic disorders including poliosis, vitiligo, alopecia, and central nervous system and auditory signs [1]. Although the etiology of VKH syndrome remains unclear, several studies have suggested that an autoimmune response against melanocytes or tyrosinase family proteins may play a key role in this disease [2,3]. Genetic predisposition is an important element as evidenced by familial cases [3] and a strong association with the human leukocyte antigen (HLA) system [4,5], especially HLA-DR4 and HLA-DRw53. However, the association between VKH syndrome and the HLA system does not fully explain the genetic risk for this disease. Moreover, little is known about the association of non-HLA genetic factors with VKH syndrome. Therefore, studies on the disease association with certain genes involved in the immune response may highlight the genetic background of VKH syndrome.

Programmed cell death 1 (PD-1), originally identified as a molecule linked to in vitro induction of apoptotic cell death in murine lymphoid cell lines [6], is a member of the cluster of differentiation (CD)28/B7 family. PD-1 contains an immunoreceptor tyrosine-based inhibiting motif (ITIM) and an immunoreceptor tyrosine-based switch motif (ITSM) [7] and is transcriptionally induced in activated T cells, B cells, and myeloid cells [8,9]. As an immunoinhibitory receptor, PD-1 has been shown to inhibit lymphocyte activation and cytokine production after interacting with its ligands, PD-L1 (B7-H1) and PD-L2 (B7-DC) [10-12]. The human gene encoding PD-1, i.e., *PDCD1*, is located on chromosome 2q37. Studies on *PDCD1* polymorphisms have demonstrated its association with several autoimmune diseases including systemic lupus erythematosus (SLE) [13-17], rheumatoid arthritis (RA) [18], type I diabetes (TID) [19], multiple sclerosis (MS) [20], ankylosing spondylitis (AS) [21], and Graves' disease [22],  although there are also some conflicting results [23-27]. Our present study was designed to investigate the association of *PDCD1* single nucleotide polymorphisms (SNPs) including PD-1.3, PD-1.5, and PD-1.6 with VKH syndrome in a Chinese Han population.

## Methods

### Patients and healthy controls

Two hundred and forty-seven VKH patients with an average age at onset of 33.6 years (109 female and 138 male) and 289 age-, sex-, ethnically-matched healthy controls with an average age of 35.4 years (129 female and 160 male) were enrolled in this study. All patients were recruited from the Uveitis Study Center of the Sun Yat-sen University (Guangzhou, China) and the First Affiliated Hospital, Chongqing Medical University (Chongqing, China) and fulfilled the First International Workshop criteria for VKH disease [28]. These new criteria mainly include bilateral diffuse choroiditis at early phage and sunset glow funds and nummular chorioretinal depigmented scars at late phase in association with neurologic, auditory, or integumentary findings. Additionally but importantly, there is no history of penetrating ocular trauma or surgery preceding the initial onset of uveitis and there is no clinical or laboratory evidence suggestive of other ocular disease entities. The clinical characteristics of the tested VKH patients were summarized in Table 1. Controls were mainly the accompanying person or spouses of the patients. The local institutional ethics committee approved the study, and written informed consent was obtained from all the study subjects. Blood samples were collected in EDTA tubes and kept at −70 °C until use.

**Table 1:** The number and ratio of VKH patients with extra-ocular clinical features.

**Clinical features**	**VKH Patients (n=247)**	
	**n**	**%**
Neck stiffness	101	40.9%
Alopecia	81	32.8%
Poliosis	71	28.7%
Tinnitus	87	35.2%
dysacusis	54	21.9%
Scalp hypersensitivity	39	15.8%
Vitiligo	36	14.6%

### Genomic DNA extraction and genotyping

Genomic DNA was isolated from peripheral blood of patients and controls using standard proteinase K digestion and phenol-chloroform extraction. Amplification of the target DNA in *PDCD1* was performed by polymerase chain reaction (PCR). We used those primers previously determined by Ferreuris-Vidal, Velazquez-Cruz, and Kong (Table 2) [14,17,18]. A 15 μl reaction mixture, which consisted of 7.5 μl Premix Taq (Ex Taq Version; TaKaRa Biotechnology Co. Ltd., Dalian, China), 50 pmoles primers, and 0.2 μg of genomic DNA, was amplified by PCR. PCR conditions were as follows: initial denaturation at 95 °C for 5 min followed by 35 cycles of denaturation at 94 °C for 30 s, annealing at different temperatures (67 °C for PD-1.3, 60 °C for PD-1.5, and 61 °C for PD-1.6) for 30 s, extension at 72 °C for 30 s, and a final extension at 72 °C for 5 min.

**Table 2:** Primers of SNPs PD-1.3, PD-1.5, and PD-1.6 and restriction enzymes used for RFLP analysis.

**Marker name**	**SNP**	**rs number**	**Location**	**allele**	**Primers**	**Restriction enzyme**
PD-1.3	7146G/A	rs11568821	Intron 4	A	5′-CCCCAGGCAGCAACCTCAAT-3′	PstI
					5′-GACCGCAGGCAGGCACATAT-3′	
PD-1.5	7785C/T	rs2227981	Exon 5	C	5′-GTGCCTGTGTTCTCTGTGGA-3′	Pvu II
					5′-CCAAGAGCAGTGTCCATCCT-3′	
PD-1.6	8737G/A	rs10204525	3′-UTR	A	5′-TCAGAAGAGCTCCTGGCTGT-3′	Nla III
					5′-GGGGAACGCCTGTACCTT-3′	

The SNPs were genotyped by PCR-restriction fragment length polymorphism (RFLP) analysis. PCR products of PD-1.3, PD-1.5, and PD-1.6 polymorphisms were respectively digested with 2 U of PstI (Fermentas international Inc., Ontario, Canada), Pvu II (Fermentas international Inc.), and Nla III (New England Biolabs, Inc., Ontario, Canada) restriction enzymes (Table 2) in a 10 µl reaction volume overnight. Digestion products were visualized on agarose gels of appropriate concentration and stained with GoldView™ (SBS Genetech, Beijing, China). To confirm the accuracy of the method employed, randomly selected subjects (20% of all samples) were analyzed by direct sequencing (Invitrogen Biotechnology Co., Guangzhou, China). Appropriate controls (no template and known genotype) were included in each typing run. HLA-DR4 and HLA-DRw53 typing was performed using a PCR sequence specific primers (SSP) method as previously described [29].

### Statistical analysis

Hardy–Weinberg equilibrium (HWE) was tested using the χ^2^ test. Allele frequencies were estimated by direct counting. Allele and genotype frequencies were compared between patients and controls by the χ^2^ test using SPSS (version 10.0; SPSS Inc., Chicago, IL). The p values were corrected (pc) with the Bonferroni correction by multiplying with the number of analyses performed. pc<0.05 was considered significant.

## Results

### Association analysis of single nucleotide polymorphism and haplotype

Three SNPs were successfully genotyped in 247 patients with VKH syndrome and 289 healthy controls. Table 3 shows the genotype and allele distribution of these SNPs. The distribution of genotype frequencies of each SNP in the healthy controls did not show any significant deviation from the Hardy–Weinberg equilibrium. The frequency of the C allele of SNP PD-1.5 tended to be lower in VKH patients than in healthy controls (71.5% versus 78.0%, p=0.013). However, no difference was found when the Bonferroni correction was applied (pc=0.078, n=6). Similarly, although a lower frequency of SNP PD-1.5CC genotype was observed in VKH patients as compared with that in healthy controls, the difference was not statistically significant following the Bonferroni correction (p=0.034, pc=0.306, n=9). The genotype and allele frequencies of PD-1.3 and PD-1.6 in patients with VKH syndrome were not different from those in healthy controls. In particular, only the GG genotype and the G allele of PD-1.3 were identified in all patients and controls.

**Table 3:** Genotype and allele frequencies of SNPs PD-1.3, PD-1.5, and PD-1.6 in VKH patients and healthy controls.

***PDCD1* SNPs**	**VKH patients (%)**	**Healthy controls (%)**	**χ^2^**	**p**	**pc**	**Odds ratio (95%CI)**
**PD-1.3**						
Genotype						
GG	247 (100)	289 (100)	—	—	—	—
AG	—	—				
AA	—	—				
Allele						
G	494 (100)	578 (100)	—	—	—	—
A	—	—				
**PD-1.5**						
Genotype						
CC	131 (53.0)	176 (60.9)	6.786	0.034	0.306	—
CT	91 (36.8)	99 (34.3)				
TT	25 (10.1)	14 (4.8)				
Allele						
C	353 (71.5)	451 (78.0)	6.132	0.013	0.078	0.705 (0.534–0.930)
T	141 (28.5)	127 (22.0)				
**PD-1.6**						
Genotype						
AA	124 (50.2)	136 (47.1)	5.171	0.075	—	—
AG	108 (43.7)	119 (41.2)				
GG	15 (6.1)	34 (11.7)				
Allele						
A	356 (72.1)	391 (67.6)	2.413	0.12	—	1.232 (0.947–1.603)
G	138 (27.9)	187 (32.4)				

The distribution of genotype frequencies of each SNP in the healthy controls did not show significant deviation from the Hardy-Weinberg equilibrium. The frequency of the C allele and CC genotype of PD-1.5 were lower in VKH patients than in healthy controls, but the differences were not statistically significant following the Bonferroni correction. The genotype and allele frequencies of PD-1.3 and PD-1.6 in VKH patients were not different from those in healthy controls. Only the GG genotype and the G allele of PD-1.3 were identified in all patients and controls

Haplotype analysis using Haploview software showed no linkage disequilibrium in the tested three SNPs in both patients with VKH syndrome and healthy controls.

### Stratification analysis according to HLA status and clinical characteristics

The frequencies of HLA-DR4 and HLA-DRw53 were shown to be significantly increased in 231 VKH patients as compared with those in 289 healthy controls (Table 4). The allele and genotype frequencies of PD-1.3, PD-1.5, and PD-1.6 were not different between VKH patients and healthy controls when a stratification analysis was performed according to the status of HLA-DR4 and HLA-DRw53.

**Table 4:** HLA-DR4 and HLA-DRw53 distributions of patients with VKH syndrome.

**HLA**	**VKH patients (n=231)**	**Normal controls (n=289)**	**χ^2^**	**p**
HLA-DR4+	179 (77.5%)	59 (20.4%)	169.712	0
HLA-DR4-	52 (22.5%)	230 (79.6%)		
HLA-DRw53+	203 (87.9%)	193 (66.8%)	32.07	0
HLA-DRw53-	28 (12.1%)	96 (33.2%)		

The frequencies of HLA-DR4 and HLA-DRw53 were significantly increased in 231 VKH patients as compared with those in 289 healthy controls.

When the genotype frequencies were analyzed according to the clinical features, our results showed significantly lower frequencies of PD-1.5CC in VKH patients either with poliosis or with dysacusis as compared to that observed in healthy controls. Concerning the allele frequencies analyzed according to the clinical features within the VKH cohort, it was found that significantly lower frequencies of the PD-1.5C allele were associated with VKH patients with extraocular findings including alopecia, poliosis, dysacusis, and tinnitus (Table 5). Neither PD-1.3 nor PD-1.6 was found to be associated with any of the extraocular findings including alopecia, poliosis, dysacusis, tinnitus, and neck stiffness.

**Table 5:** Genotypes and alleles frequencies of PD-1.5 SNP in VKH patients and healthy controls according to the clinical features.

**Clinical features**	**PD-1.5 SNP**	**VKH patients (%)**	**Healthy controls (%)**	**χ^2^**	**p**	**pc**	**Odds ratio (95%CI)**
Alopecia	CC	37 (45.7)	176 (60.9)	9.083	0.011	0.099	
	CT	34 (42.0)	99 (34.3)				
	TT	10(12.3)	14 (4.8)				
	C	107 (66.0)	451 (78.0)	9.78	0.002	0.016	0.548 (0.375–0.801)
	T	55 (34.0)	127 (22.0)				
Poliosis	CC	27 (38.0)	176 (60.9)	15.454	0	0	
	CT	34 (47.9)	99 (34.3)				
	TT	10 (14.1)	14 (4.8)				
	C	89 (62.7)	451 (78.0)	14.328	0	0	0.473 (0.319–0.700)
	T	53 (37.3)	127 (22.0)				
Dysacusia	CC	24 (44.4)	176 (60.9)	17.172	0.0005	0.0045	
	CT	19 (35.2)	79 (34.3)				
	TT	11 (20.4)	14 (4.8)				
	C	68 (63.0)	451 (78.0)	11.213	0.0001	0.008	0.479 (0.309–0.741)
	T	40 (37.0)	127 (22.0)				
Tinnitus	CC	42 (48.3)	176 (60.9)	9.839	0.007	0.063	
	CT	33 (37.9)	99 (34.3)				
	TT	12 (13.8)	14 (4.8)				
	C	118 (67.8)	451 (78.0)	7.574	0.006	0.048	0.593 (0.408–0.863)
	T	56 (32.2)	127 (22.0)				
Neck stiffness	CC	52 (51.5)	176 (60.9)	8.142	0.017	0.153	
	CT	36 (35.6)	99 (34.3)				
	TT	13 (12.9)	14 (4.8)				
	C	140 (69.3)	451 (78.0)	6.201	0.013	0.078	0.636 (0.445–0.910)
	T	62 (30.7)	127 (22.0)				

The genotype frequencies of PD-1.5 CC in VKH patients either with poliosis or with dysacusia were lower than that observed in healthy controls. Within the VKH cohort, significantly lower frequencies of the PD-1.5 C allele were associated with VKH patients with any one of the extraocular findings including alopecia, poliosis, dysacusia and tinnitus.

## Discussion

Our results showed that a lower frequency of PD-1.5 genotype or allele was associated with certain extraocular findings within the VKH cohort. However, PD-1.3 and PD-1.6 polymorphism differences were identified between VKH patients and healthy controls.

VKH syndrome is a multifactorial disease that may result from interactions among susceptibility genes, environmental factors, and immunological responses. The association of HLA-DR4 and HLADRw53 with VKH syndrome has been reported in the Chinese [4] and Japanese [5] populations. Our present study confirmed these observations. The non-HLA genetic background of the disease has not been completely understood. It is reported that there are no genetic susceptibilities for the tyrosinase gene family and interferon-γ in Japanese VKH patients [30,31]. Recently, we reported an association of cytotoxic T lymphocyte-associated antigen-4 (*CTLA-4*) genetic polymorphisms with the susceptibility to VKH syndrome in a Chinese population [32]. PD-1 is an immunoglobulin (Ig) superfamily member related to CD28 and CTLA-4. One hundred and thirty-five SNPs (found in the NCBI-Entrez SNP database) have been identified in the human *PDCD1* region. Several studies showed that polymorphisms of PD-1.3, PD-1.5, and PD-1.6 were associated with autoimmune diseases [13-22], although inconsistent results were also reported [23-27]. In this study, we tested whether the polymorphisms of these three SNPs were associated with VKH syndrome in Han Chinese patients.

To ensure the analysis results, the following attempts were made. First, all genotype distributions of three SNPs in healthy controls were tested and found to be in Hardy–Weinberg equilibrium. Second, the controls and patients were strictly matched according to the places where they were born to exclude the possible influence of stratification of the population. VKH patients of Chinese Han descendents were also strictly selected in this study to avoid the influence of gene background. Third, a total of 247 VKH patients and 289 age-, sex-, ethnically-matched healthy controls were used in this study, and the number of tested samples was large enough to avoid a bias of the results. Finally, direct sequencing was performed to validate the genotype findings.

As genetic interaction between HLA genes and non-HLA genes may influence the susceptibility to autoimmune disease, a stratification analysis was performed according to the status of HLA-DR4 and HLA-DRw53. Unexpectedly, no association with *PDCD1* was observed in HLA-DR4 and HLA-DRw53 positive or negative VKH patients. As VKH syndrome affects not only the eye but also skin and hair, we further analyzed the association of polymorphisms of three SNPs with extraocular findings in VKH patients. Lower frequencies of genotype and allele of PD-1.5 were observed in VKH patients with any of the extraocular findings including alopecia, poliosis, dysacusis, or tinnitus. These results seem to suggest that the genotype and allele of PD-1.5 may possibly protect VKH patients from extraocular findings.

Several previous studies have shown that there is a large variation in the frequencies of *PDCD1* polymorphisms among different ethnic groups. In the present study, we found a higher frequency of the PD-1.5C allele and PD-1.6A allele in Chinese (78%, 67%, respectively) than in Swedish (60%, 8%, respectively), European American (57%, 11%, respectively), Mexican (54%, 39%. respectively) and African American individuals (42%, 48%, respectively) [13]. A higher frequency of the PD-1.3A allele was reported in the European population (5%–12%) compared to that observed in the Mexican and African American populations (2%–7%) [13,16] Interestingly, we did not detect the PD-1.3A allele in either the healthy controls or the VKH patients in this study, which is consistent with earlier results in healthy Hong Kong Chinese [18] and Japanese populations [27]. With regards to the association of PD-1.5 polymorphism with autoimmune disease, a study by Lee et al. [21] showed that a PD-1.5 polymorphism was positively associated with ankylosing spondylitis in Korean patients. Kong and Iwamoto found that a polymorphism of PD-1.5 was not associated with rheumatoid arthritis in Hong Kong Chinese [18] and Japanese [27] patients.

In conclusion, this study showed that all genotype distributions of PD-1.3, PD-1.5, and PD-1.6 in Chinese Han healthy controls were in Hardy–Weinberg equilibrium. Polymorphisms of PD-1.3 and PD-1.6 were not associated with the susceptibility to VKH syndrome in a Chinese Han population. However, a genotype or allele of PD-1.5 was negatively associated with VKH patients having accompanying extraocular findings. It is not clear whether this association is present in other ethnic VKH patients and whether other *PDCD1* polymorphisms are associated with the susceptibility to VKH syndrome. More studies are needed to clarify these issues within the VKH cohort.
